# Integrated monitoring of enveloped viruses in hospital environments: Detection, persistence, and implications for infection control

**DOI:** 10.1371/journal.pone.0345644

**Published:** 2026-04-30

**Authors:** Yalda Hashempour, Mohammad-Ali Zazouli, Nematallah Jaafarzadeh, Reza Valadan, Soheila Golchin, Zabihollah Yousefi, Ramzan-Ali Dianati, Ali Atamaleki, Fatemeh Mortezazadeh, Atefeh Jabari

**Affiliations:** 1 Department of Environmental Health Engineering, School of Public Health, Health Sciences Research Center, Mazandaran University of Medical Sciences, Sari, Iran; 2 Department of Environmental Health Engineering, School of Public Health, Health Sciences Research Center, Mazandaran University of Medical Sciences, Sari, Iran; 3 Toxicology Research Center, Medical Basic Sciences Research Institute, Ahvaz Jundishapur University of Medical Sciences, Ahvaz, Iran; 4 Department of Immunology, School of Medicine, Mazandaran University of Medical Sciences, Sari, Iran; 5 Non-Communicable Disease Institute, Imam Khomeini Hospital, Sari, Iran; 6 Department of Environmental Health Engineering, School of Public Health, Mazandaran University of Medical Sciences, Sari, Iran; 7 Department of Environmental Health Engineering, School of Public Health, Mazandaran University of Medical Sciences, Sari, Iran; 8 Department of Environmental Health Engineering, School of Public Health and Safety, North Khorasan University of Medical Sciences, Bojnord, Iran; 9 Department of Environmental Health Engineering, School of Health, Tehran University of Medical Sciences, Tehran, Iran; 10 Department of Environmental Health Engineering, School of Public Health, Mazandaran University of Medical Sciences, Sari, Iran; Waseda University: Waseda Daigaku, JAPAN

## Abstract

Enveloped viruses, including coronaviruses, influenza viruses, and others, pose significant public health risks due to their ability to persist in various environments. This study investigates the persistence of enveloped viruses, particularly SARS-CoV-2, in three key environments: air, surfaces, and wastewater, with a focus on hospital settings. We present a systematic review of the literature on the environmental persistence of these viruses, complemented by a case study conducted in two reference hospitals in northern Iran. A total of 72 wastewater samples, 46 air samples, and 92 surface samples were collected from COVID-19 wards and analyzed using RT-PCR for the presence of viral RNA. Results indicate notable viral persistence, with detection rates of 21.74% in air samples, 26.09% in surface samples, and 63.89% in wastewater samples. This indicates substantial viral shedding from infected patients, particularly in influent samples, where treatment inefficiency contributed to higher detection. It is important to note that RT-PCR detects viral RNA, which does not necessarily indicate infectivity; however, its presence highlights routes of potential transmission and the need for vigilant environmental management. The findings highlight the critical need for enhanced environmental monitoring and the strict implementation of effective disinfection protocols to mitigate the risk of viral transmission in healthcare settings. The persistence of SARS-CoV-2 in hospital environments underscores the importance of addressing fomite transmission and optimizing ventilation strategies. Overall, this comprehensive approach provides valuable insights into the behavior of enveloped viruses in diverse settings and emphasizes the necessity of integrated surveillance strategies to aid infection control efforts and protect public health.

## 1. Introduction

Hospitals are high-risk environments for viral transmission due to the concentration of infected individuals, high-touch surfaces, and the potential for aerosol generation during medical procedures [1,2]. Enveloped viruses, characterized by their lipid bilayer envelope, include some of the most significant human pathogens, such as coronaviruses (e.g., SARS-CoV-2), influenza viruses, and herpesviruses [3,4]. These viruses are known for their ability to persist in various environments, including air, surfaces, and water, making them a persistent threat to public health, particularly in healthcare settings [5,6]. The COVID-19 pandemic has underscored the importance of understanding the environmental persistence of enveloped viruses, as SARS-CoV-2 has been detected in hospital air, on surfaces, and in wastewater [7]. The persistence of these viruses in the environment is influenced by several factors, including temperature, humidity, surface material, and the presence of organic matter [8]. Understanding these factors is critical for developing effective strategies to mitigate viral transmission, especially in high-risk settings such as hospitals [9,10].

The persistence of enveloped viruses in the environment has been extensively studied, particularly in the context of the COVID-19 pandemic. Research has shown that SARS-CoV-2 can remain viable in aerosols for hours, with survival influenced by humidity, temperature, and ventilation [11,12]. Studies have also demonstrated that enveloped viruses can persist on surfaces for extended periods, with longer survival on non-porous surfaces such as plastic and stainless steel [13,14]. In wastewater, enveloped viruses, including SARS-CoV-2, can persist for days, with detection possible even after treatment [15,16].

The detection of SARS-CoV-2 in hospital environments has raised concerns about the potential for environmental transmission. Studies have shown that SARS-CoV-2 RNA can be detected in the air of hospital wards, particularly in poorly ventilated areas [17,18]. High-touch surfaces such as bed rails, doorknobs, and medical equipment have also been found to be frequently contaminated with SARS-CoV-2 RNA [19]. Wastewater-based epidemiology (WBE) has emerged as a valuable tool for monitoring viral outbreaks, with SARS-CoV-2 RNA detected in wastewater from hospitals and communities [20].

Despite these advances, there are still significant gaps in our understanding of the persistence of enveloped viruses in the environment. For example, the factors influencing the survival of enveloped viruses in different environments are not fully understood. Additionally, the effectiveness of disinfection protocols in reducing viral persistence in healthcare settings needs further investigation. There is also a need for more research on the role of environmental monitoring in preventing viral transmission, particularly in resource-limited settings [21,22].

While significant progress has been made in understanding the persistence of enveloped viruses in the environment, several knowledge gaps remain. First, most studies have focused on SARS-CoV-2, with limited research on other enveloped viruses such as influenza viruses and herpesviruses. This limits our understanding of the general behavior of enveloped viruses in the environment. Second, there is a lack of comprehensive studies that integrate data from multiple environments (air, surfaces, and wastewater) to provide a holistic view of viral persistence. Third, the effectiveness of disinfection protocols in reducing viral persistence in healthcare settings needs further investigation. Finally, there is a need for more research on the role of environmental monitoring in preventing viral transmission, particularly in resource-limited settings.

Therefore, this study addresses these knowledge gaps by focusing on the persistence of enveloped viruses, particularly SARS-CoV-2, in air, surface, and wastewater samples in hospital environments providing real-world data on the persistence of the viruses in healthcare environments. To contextualize the findings of the case study, this research also includes a systematic review of the literature on the persistence of enveloped viruses in air, surfaces, and wastewater. By integrating data from air, surfaces, and wastewater, this study offers a comprehensive understanding of viral persistence in healthcare settings. This approach is innovative because it not only identifies the factors influencing viral persistence but also evaluates the effectiveness of current disinfection protocols and provides actionable recommendations for improving environmental monitoring and infection control practices.

## 2. Materials and methods

### 2.1. Case Study: Hospital Environments

#### 2.1.1. Description of the Hospitals.

Mazandaran Province, with its administrative center in Sari, is one of the most important regions in northern Iran for the identification and treatment of respiratory virus cases. Patients are admitted to several medical centers, with Imam-Khomeini Hospital in Sari and Razi Hospital in Qaem-Shahr being the primary facilities for hospitalization and treatment of respiratory virus cases. The geographical coordinates of the hospital locations are as follows: Imam-Khomeini Hospital in Sari (36.5659° N, 53.0586° E) and Razi Hospital in Qaem-Shahr (36.4631° N, 52.8600° E).

At the time of sampling (between May and August 2022), Mazandaran Province was experiencing the fifth wave of the COVID-19 outbreak, classified as a “red zone” due to high transmission rates. Epidemiological investigations identified the Beta variant (lineage B.1.351) as the dominant strain in the region during this period.

Imam-Khomeini Hospital in Sari is a 410-bed facility with 25 wards. Wastewater from all wards is directed to Wastewater Treatment Plant A (WWTP A). Razi Hospital in Qaem-Shahr is a 253-bed facility with 15 wards, and its wastewater is treated at Wastewater Treatment Plant B (WWTP B). Both treatment plants operate using an extended aeration-activated sludge process. The treatment units at Imam-Khomeini Hospital include screening, aeration, sedimentation, and a chlorination basin, with a flow rate of 130 m^3^/day. Razi Hospital’s treatment plant consists of a selector pond, aeration, sedimentation, chlorination, and a digester basin, with a flow rate of 140 m^3^/day. Disinfection at both plants is achieved using perchlorate powder.

During the sampling period, 1,120 patients with suspected COVID-19 were hospitalized in Mazandaran Province based on clinical diagnosis, of which 198 were admitted to Intensive Care Units (ICUs) due to acute respiratory symptoms. Sari, with a population of 309,820, reported 30–58 COVID-19 cases, while Qaem-Shahr, with a population of 204,953, reported 47–68 cases during the same period.

This study did not involve human participants, human specimens, or personal data. All samples were collected from environmental sources (air, surfaces, and wastewater) in hospital settings. Therefore, ethics approval and consent to participate were not applicable.

#### 2.1.2. Pre-sampling measures.

Before commencing each sampling session, all test kits, which had been previously autoclaved, were disinfected using a 70% alcohol solution in accordance with the protocol established by the Centers for Disease Control and Prevention (Atarod et al., 2022). Dulbecco’s Modified Eagle’s Medium (DMEM) liquid was then prepared in a sterile culture medium suitable for coronavirus growth and placed in impregnated glass tubes (Nagle et al., 2022). These prepared impingers, containing the culture medium, were stored at a temperature below 4 °C, and their lids were securely sealed until sampling began.

#### 2.1.3. Collection, storage, and transfer of wastewater samples.

In this comprehensive study, a total of 72 grab wastewater samples (36 influent and 36 effluent) were collected through weekly sampling over an 18-week period (May to August 2022) from two hospital wastewater treatment plants, providing sufficient temporal coverage to capture fluctuations in viral shedding during the study period. As a precautionary measure, all collected wastewater samples were transferred into 100 mL autoclaved plastic bottles and transported to the laboratory within two hours using cold packs (4 °C). The samples were stored at −80 °C until analyzed using reverse transcription-polymerase chain reaction (RT-PCR). The electronegative membrane-vortex (EMV) method, as described by Haramoto et al. (2013) and adapted by Wilhelm et al. (2022) [20], was employed to concentrate viral particles from the wastewater samples. A 100 mL sample was spiked with 1 mL of 2.5 mol/L MgCl₂. The sample was then filtered through a cellulose-ester membrane (0.8 μm pore size, 47 mm diameter; Merck Millipore, Billerica, MA, USA). The membrane was placed in a plastic tube containing 10 mL of elution buffer and mixed vigorously using a ball-shaped stirrer. The eluate was collected in a new 50 mL plastic tube. This process was repeated with 5 mL of elution buffer until a final volume of approximately 15 mL was achieved. The eluate was clarified by centrifugation (10 minutes, 2000 × g, 4 °C), and the supernatant was further filtered through a 0.45 μm membrane filter. Finally, the filtrate was concentrated using Centriprep YM-50 ultrafiltration to achieve a higher viral concentration. All wastewater samples were analyzed in duplicate, and the mean Ct value was reported for positive samples. Samples with discordant results (positive/negative between duplicates) were re-analyzed in triplicate, and the consensus result was reported.

#### 2.1.4. Active air sampling in hospitals.

Active air sampling was conducted using impingers, a highly efficient method for collecting indoor air samples by impinging an air stream into a liquid medium [23]. This device included 1) SKC sampling pumps (AirChek® Serie) with suction power and flow rate of 5 L.min^-1^; 2) an air flow calibrator (with CalChek Cat. No. 375-0550N); 3) the glass impinge; 4) the glass impinge trap; 5) the stainless steel holder for area sampling; and 6) trap sorbent to protect the pump from both organic and inorganic vapors. The sampling pumps were equipped with polytetrafluoroethylene (PTFE) membrane filters (37 mm diameter, 0.3 μm pore size), which were calibrated before each sampling session using an electronic calibrator (Bios Defender 510 H, USA) [2].

Air sampling was conducted in specialized wards for confirmed COVID-19 cases, COVID-19 ICUs, and administrative departments. The sampling pumps were positioned at a height of 1.5 to 2 meters above the ground, corresponding to the respiratory level of individuals, and at horizontal distances of 0.5, 1, 2, and 5 meters from the beds of confirmed COVID-19 patients. In the CT scan ward, sampling was performed at the head and body locations, as well as at a height of 1.5 meters above the ground.

During the sampling period, natural ventilation was absent, and mechanical ventilation systems were turned off. The sampling areas were not disinfected prior to sampling. To maintain indoor air temperature in the ICU during spring, a split air conditioner was installed at the entrance of the ward.

After identifying the sampling points, the inlet and outlet ports of the impingers were unsealed, and the inlet was connected to the SKC sampling pumps. The pumps, pre-adjusted and calibrated, were allowed to operate for 4 hours. Ambient temperature and humidity were measured using an HD50 hygrometer (KIMO, France), and particulate matter mass concentration (PMmass) in the indoor air was quantified using a portable Grimm 108 device (Grimm Aerosol Technik, Germany) for weighing and counting dust particles.

At the end of the 4-hour sampling period, the pumps were turned off, and the impingers containing the culture medium were disconnected. The inlet and outlet ports were resealed with sterile caps, and the samples were refrigerated at 4 °C before being promptly transferred to the virology reference laboratory for viral detection.

Air sampling at each distance and location was performed once due to logistical constraints associated with 4-hour sampling duration and limited impinger availability. However, the extended sampling time (4 hours) integrated viral particles over time, providing a representative measure of airborne contamination. Additionally, sampling was repeated on different days (three separate sessions) at selected locations to assess temporal variability.

#### 2.1.5. Fomite sampling in hospitals.

In this study, the spread of the virus was measured in both Infection wards (housing confirmed COVID-19 cases) and non-Infection wards as part of a highly efficient sampling approach. Three Infection wards—dedicated to COVID-19 patients, including the special ward, ICU, and CT scan ward—and one non-infectious ward (the working room of the hospital’s environmental health department) were selected for sampling. High-risk environments and high-touch surfaces were identified as sampling locations in accordance with World Health Organization (WHO) guidelines [24].

Routine cleaning and disinfection of the sampling locations in both hospitals were performed twice daily using Septi Surface solution, which contains 500 mg/L chlorine (Behban Pharmed Lotus®, Iran). The active ingredients of this product—ethanol, chlorhexidine digluconate, and alkyl dimethyl benzyl ammonium chloride—are effective against a wide range of bacteria, viruses, and fungi. Cleaning rounds were conducted in the morning (9:00–10:30 AM) and afternoon (2:30–4:00 PM). Sampling was carried out before the morning cleaning round to avoid interference from disinfection activities.

A total of 92 samples (46 per hospital) were collected from high-touch surfaces and critical points in various parts of the hospitals. Sampling was performed using nylon flocked swabs, which were removed from their packaging and moistened with 2 mL of virus transport medium (serum-free DMEM, Biosera, UK). The swabs were rotated with sufficient pressure in multiple directions over a minimum surface area of 25 cm^2^. The swabs were then transferred to tubes containing 200 mL of twice-distilled sterile water mixed with antibiotic, protein stabilizer, and buffer solution.

The swab samples were labeled, stored at 4 °C, and transported to the virology laboratory for analysis. Control samples were collected alongside experimental samples to ensure accuracy. After processing, the samples were tested using PCR, and results were reported as positive or negative for viral presence.

Each high-touch surface was sampled at a single time point during each weekly visit. However, to capture temporal variation in contamination levels, sampling was repeated weekly over the 18-week study period at selected high-touch surfaces (bed rails, doorknobs, bedside tables) in COVID-19 wards and ICUs.

#### 2.1.6. RNA extraction and real-time PCR.

All collected samples were immediately immersed in viral transfer medium and stored at −80 °C until analysis. Viral RNA was extracted using a commercial viral RNA extraction kit (Viragene, Iran) following the manufacturer’s instructions. Briefly, 200 μL of sample was mixed with 400 μL of lysis buffer containing guanidine isothiocyanate and incubated at room temperature for 10 minutes. After adding 600 μL of binding buffer (ethanol-based), the mixture was passed through a silica membrane column by centrifugation at 8,000 × g for 1 minute. The column was washed twice with 500 μL of wash buffers, and RNA was eluted in 50 μL of RNase-free water by centrifugation at 10,000 × g for 2 minutes. The extracted RNA was stored at −80 °C until amplification.

Real-time reverse transcription-polymerase chain reaction (RT-PCR) was performed to detect SARS-CoV-2RNA targeting two viral genes: the nucleocapsid (N) gene and the RNA-dependent RNA polymerase (RdRp) gene, as described by Atarod et al. (2022) [23] with minor modifications.

#### 2.1.7. Quality control measures.

To ensure the reliability and validity of the results, several quality control measures were implemented throughout the sampling and analysis process. For each sampling session, field blank samples (sterile containers exposed to the sampling environment without sample collection) were transported alongside actual samples to monitor potential cross-contamination during handling and transport. A total of 10 field blanks (5 for each hospital) were collected and analyzed; all tested negative for SARS-CoV-2 RNA.

For RT-PCR analysis, nuclease-free water was used as a negative control in each PCR run to rule out reagent contamination. Additionally, for surface sampling, sterile swabs moistened with virus transport medium were processed as negative controls (one per sampling session). All negative controls consistently yielded negative results.

Known SARS-CoV-2 RNA standards (synthetic controls targeting N and RdRp genes) were included in each RT-PCR run to validate assay performance and confirm successful amplification. Positive controls consistently showed expected Ct values (range: 28–32).

A subset of samples was analyzed in duplicate to assess reproducibility: 10% of wastewater samples (7 samples), 15% of air samples (7 samples), and 10% of surface samples (9 samples). The coefficient of variation (CV) for Ct values ranged from 1.2% to 4.8%, indicating excellent reproducibility.

All sampling equipment was autoclaved or disinfected with 70% ethanol before use. Personnel wore appropriate personal protective equipment (PPE) including gloves, masks, and gowns, and changed gloves between sampling sites to prevent cross-contamination. Sampling was consistently performed before morning cleaning rounds to avoid interference from disinfection activities. The primer and probe sequences were as follows:

**N gene assay:**
  Forward primer: 5’-GGGGAACTTCTCCTGCTAGAAT-3’
  Reverse primer: 5’-CAGACATTTTGCTCTCAAGCTG-3’
  Probe: FAM-5’-TTGCTGCTGCTTGACAGATT-3’-BHQ1
**RdRp gene assay:**
  Forward primer: 5’-GTGARATGGTCATGTGTGGCGG-3’
  Reverse primer: 5’-CARATGTTAAASACACTATTAGCATA-3’
  Probe: VIC-5’-CAGGTGGAACCTCATCAGGAGATGC-3’-BHQ1

Each 20 μL reaction contained 5 μL of RNA template, 10 μL of 2X Master Mix (One-Step RT-PCR Master Mix, containing reverse transcriptase and Taq polymerase; Viragene, Iran), 0.5 μL of each primer (10 μM), 0.25 μL of probe (10 μM), and nuclease-free water to reach the final volume.

Thermal cycling was performed on a Rotor-Gene Q real-time PCR cycler (Qiagen, Germany) under the following conditions: reverse transcription at 50°C for 15 minutes, initial denaturation at 95°C for 3 minutes, followed by 45 cycles of denaturation at 95°C for 10 seconds and combined annealing/extension at 55°C for 30 seconds. Fluorescence signals (FAM for N gene, VIC for RdRp gene) were collected at the end of each annealing/extension step.

A sample was considered positive if it met both of the following criteria: (1) cycle threshold (Ct) value ≤ 38 for at least one target gene, and (2) exponential amplification curves with typical sigmoidal shape. Samples with Ct values between 38 and 40 were re-analyzed in duplicate; if at least one replicate showed Ct ≤ 40 with exponential amplification, the sample was reported as weakly positive. Samples with Ct > 40 or no amplification were considered negative. For quality assurance, each run included no-template controls (nuclease-free water) and positive controls (synthetic SARS-CoV-2 RNA).

Given the exploratory and descriptive nature of this environmental surveillance study, descriptive statistics (frequencies and percentages) were used to summarize the detection rates of SARS-CoV-2 RNA across different sample types and locations.

### 2.2. Systematic review

#### 2.2.1. Systematic review methodology.

**Fig 1:**
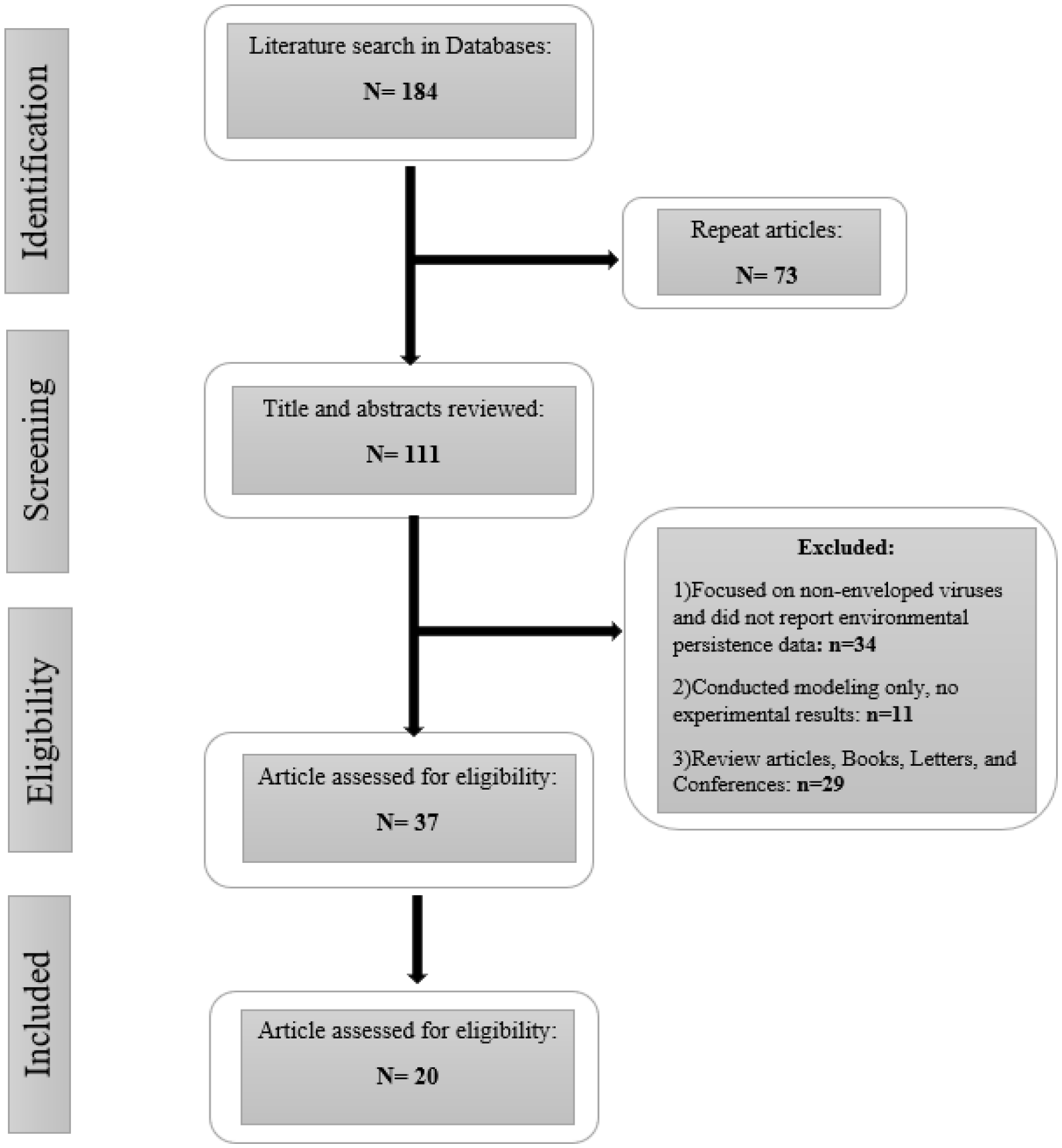
PRISMA flow diagram.

A systematic review was conducted to identify and synthesize studies reporting the persistence of enveloped viruses in air, surfaces, and wastewater environments. The review was performed in accordance with the PRISMA 2020 guidelines to ensure transparent and comprehensive reporting. The study selection process is presented in Fig. 1 (PRISMA flow chart), detailing the number of records identified, screened, included, and excluded with corresponding reasons.

#### 2.2.2. Inclusion and exclusion criteria.

Studies were included if they investigated the persistence, survival, or detection of enveloped viruses such as SARS-CoV-2, influenza viruses, or other coronaviruses in environmental matrices (air, surfaces, or wastewater), were published in English between 1995 and September 2025, and reported experimental or field-based data. Studies were excluded if they were review articles, conference abstracts without full data, modeling-only studies without empirical validation, or lacked quantitative information on environmental persistence.

#### 2.2.3. Information sources.

The literature search was conducted across PubMed, Scopus, ScienceDirect, and Web of Science databases, complemented by manual screening of reference lists and relevant gray literature. The last search was completed on September, 2025.

#### 2.2.4. Search strategy.

Search strategies were constructed using combinations of MeSH terms and keywords related to enveloped viruses and environmental persistence. A representative search strategy used for PubMed was as follows: (“enveloped virus” OR “SARS-CoV-2” OR “influenza virus” OR “coronavirus” OR “HcoV” OR “TGEV” OR “MHV” OR “Enterovirus” OR “Rhinovirus” OR “Respiratory Syncytial Virus” OR “Hepatitis” OR “HIV” OR “Adenovirus”) AND (“environmental persistence” OR “surface contamination” OR “airborne transmission” OR “wastewater-based epidemiology” OR “viral stability”). Equivalent strategies were adapted for PubMed, Scopus, ScienceDirect, and Web of Science using Boolean operators and database-specific syntax. Filters were applied to restrict results to English-language studies published between 1995 and 2025.

#### 2.2.5. Study selection and data extraction.

All identified records were imported into a reference management software, and duplicates were removed prior to screening. Two reviewers independently screened titles and abstracts for relevance, followed by full-text assessments to confirm eligibility. Discrepancies were resolved through discussion and consensus. Extracted data included virus type, environmental matrix (air, surface, or wastewater), detection method (RT-qPCR, culture-based assay, or plaque assay), persistence duration, environmental parameters such as temperature, humidity, and pH, and key study findings.

#### 2.2.6. Outcomes.

The primary outcome of interest was viral persistence, expressed as survival time or reduction in viral titer within different environmental matrices. Secondary outcomes included environmental and physicochemical factors influencing persistence such as temperature, humidity, organic matter, and pH as well as the detection and quantification methods used in each study.

#### 2.2.7. Risk of bias assessment.

The methodological robustness and potential sources of bias in the included studies were systematically evaluated using a modified version of the Joanna Briggs Institute (JBI) Critical Appraisal Checklist for Systematic Reviews and Research Syntheses (Aromataris and Munn, 2020). This tool comprises eleven criteria designed to assess the rigor of the study design, reliability of data collection, and transparency of reporting. Each item was rated as “Yes” (2 points), “Unclear” (1 point), or “No” (0 points), producing an overall quality score ranging from 0 to 22.

Two independent reviewers performed the assessment to minimize subjective bias, and any differences in scoring were resolved through mutual discussion and consensus. Studies achieving a total score of 18 or above were classified as high quality (low risk of bias), those scoring between 12 and 17 as moderate quality (moderate risk of bias), and those below 12 as low quality (high risk of bias).

The results of this evaluation provided a comprehensive overview of the methodological soundness of the included studies and helped identify areas where bias may have affected the reliability of the findings. A detailed summary of the quality assessment for all included studies is provided in the Supplementary Materials (S1 Table).

## 3. Results and discussion

### 3.1. Case study

#### 3.1.1. Wastewater based epidemiology (WBE).

The detection of SARS-CoV-2 RNA in hospital wastewater indicates the presence of the virus in the digestive system and feces of infected patients [21,25]. Based on Table 1, out of 72 wastewater samples collected from two WWTPs, 47 (65.28%) tested positive for SARS-CoV-2 RNA. Positive ratios were 61.11% (22/36) for WWTP A and 69.44% (25/36) for WWTP B. All influent samples from both WWTPs were positive during the sampling period (May-August 2022), while effluent samples showed variable detection rates. In WWTP A, four effluent samples tested positive (two in May and two in June), but none were detected in July or August, indicating improved treatment efficiency. In WWTP B, effluent samples were positive only in May (27.78% or 5/18) and late August (11.11% or 2/18), with no detections between June and early August.

**Table 1:** Detection of new coronavirus RNA in wastewater samples along with characteristics of hospital wastewaters.

Location	Parameters	Sampling time																	
		1 May	8 May	15 May	22 May	29 May	5 Jun	12 Jun	19 Jun	26 Jun	3 Jul	10 Jul	17 Jul	24 Jul	31 Jul	7 Aug	14 Aug	21 Aug	28 Aug
WWTP A	Virus in Inf.	+	+	+	+	+	+	+	+	+	+	+	+	+	+	+	+	+	+
	Virus in Eff.	+	+	–	–	–	–	–	+	+	–	–	–	–	–	–	–	–	–
	No. of Patient	55	57	50	52	48	47	47	58	58	45	42	40	38	37	35	32	30	30
	T (ºC)	20	21	21	22	25	26	25	27	28	28	27	29	25	26	28	29	27	29
	TC (MPN/100 mL)	>1100	>1100	25	65	45	35	75	>1100	>1100	110	150	75	85	120	65	140	75	75
	FC (MPN/100 mL)	>1100	1100	<3	55	25	10	65	>1100	>1100	68	52	11	54	75	45	70	65	48
	pH	7.9	7	7.4	7.5	7	7.8	7.5	7.5	7	7.2	7.8	7.2	7	7.5	7.9	7.8	7.6	7.5
	DO (mg/L)	6	6	6	7	6	7	7	6	6	7	6	7	6	7	7	6	6	6
	BOD (mg/L)	61.8	51.9	10	10.5	10.2	9.5	9	62	52.6	10.2	10.5	9	9.8	10.2	10.1	9.8	9.9	10
	COD (mg/L)	88.7	78.9	15.2	16.2	16	14.4	13.7	98.2	79.5	15.5	16.1	13.6	15	15.6	15.5	15.2	15.1	15.4
	TSS (mg/L)	10	10.5	9	8.5	8	7.9	8.5	10.7	11	9	9.5	8.9	7	7.5	7.6	8	7	7.5
	Chloride (mg Cl^-^/L)	230	241	225	205	210	189	175	235	245	165	145	163	174	165	150	163	145	136
	NH_3_ (mg/L)	1.2	1	1.1	1.1	0.4	0.9	1	1.6	0.7	0.9	1.2	1.5	0.7	0.9	1.1	1.1	0.7	1.2
	NO_3_ (mg/L)	176	185	135	125	117	123	116	169	174	123	114	111	109	114	123	120	117	110
	Resi. Chlo. (mg/L)	0	0	1.5	1	2	1.7	1.5	0	0	0.7	1.2	1.5	2.1	2	1.8	1.7	1.5	1
	TP (mg/L)	7.5	7.2	4.2	5.5	2.7	2.5	4.2	6.7	7.2	2.1	2	2.7	3.2	3.5	2.7	4.3	5	3.8
WWTP B	Virus in Inf.	+	+	+	+	+	+	+	+	+	+	+	+	+	+	+	+	+	+
	Virus in Eff.	+	+	+	+	+	–	–	–	–	–	–	–	–	–	–	–	+	+
	No. of Patient	62	65	66	65	67	50	52	55	47	51	55	52	52	45	47	55	67	68
	T (ºC)	20	22	23	20	24	27	25	27	27	27	26	29	25	26	28	29	27	29
	TC (MPN/100 mL)	>1100	>1100	>1100	>1100	>1100	235	175	245	170	225	245	220	252	245	265	235	>1100	>1100
	FC (MPN/100 mL)	>1100	>1100	>1100	>1100	>1100	460	450	550	750	470	570	490	650	660	510	570	>1100	>1100
	pH	7.5	7.2	7.1	7.2	7	7.8	7.7	7.1	7.2	7.2	7.8	7.5	7	7.8	7.9	7.2	7.1	7.5
	DO (mg/L)	4.6	6.2	6.7	4.7	6.2	5.7	6.5	4.6	4.8	7.2	6	7.5	6.9	7	6.5	6.2	6.4	6.1
	BOD (mg/L)	71.8	71.9	55	60.5	60.2	45.5	39	42	42.6	40.2	30.5	49	39.8	40.2	40.1	49.8	69.9	70
	COD (mg/L)	110	109	87.5	98	95.5	70.1	60.1	64.5	66	62.7	65.5	89	75.2	65.8	69	75	110	125
	TSS (mg/L)	68.8	61.9	52	56.5	55.2	42.5	32	40	40	40	25	45	34.8	35.5	36	45	66.6	67
	Chloride (mg Cl^-^/L)	250	261	245	225	250	199	195	255	268	205	489	188	198	185	172	191	175	145
	NH_3_ (mg/L)	2.2	2	2.1	2.1	1.4	1.9	1.5	1.9	1.7	1.9	1.9	2.1	1.7	1.9	2.1	2.1	1.7	1.9
	NO_3_ (mg/L)	186	199	155	145	147	133	126	175	174	144	124	111	119	124	135	120	119	120
	Resi. Chlo. (mg/L)	0	0	0	0	0	1.1	1.0	1.2	0.9	0.9	1.5	1.1	1.1	1	1.8	1.5	0	0
	TP (mg/L)	7.9	7.8	7.8	7.9	6.7	3.5	5.2	5.7	6.2	4.1	5.5	2.7	3.2	4.7	5.7	5.3	5	5.8

Note: WWTP A = Wastewater Treatment Plant of Imam-Khomeini Hospital; WWTP B = Wastewater Treatment Plant of Razi Hospital; Inf. = wastewater influent; Eff. = wastewater effluent; T = wastewater Temperature; No. of Patient = Number of hospitalized COVID-19 patients; TC = Total Coliform; FC = Fecal Coliform; Resi. Chlo = Residual chlorine in wastewater; and TP = Total phosphorous (mg/L).

The persistence of SARS-CoV-2 RNA in wastewater is influenced by factors such as organic pollutants, pH, temperature, and disinfection practices [26]. In our study, positive samples were observed at temperatures ranging from 20–28°C, suggesting that temperature alone did not significantly affect viral RNA detection. However, other studies have highlighted the role of temperature in viral stability, with lower temperatures favoring longer persistence [27,28].

Chlorination was used as a pre-treatment step before wastewater discharge. Residual chlorine levels were critical; when absent, effluent samples tested positive for SARS-CoV-2 RNA. Zhang et al., (2020) demonstrated that free chlorine at 6.5 mg/L with 1.5 hours of contact time effectively eliminated SARS-CoV-2 RNA (Zhang et al., 2020). However, RNA persistence in chlorinated wastewater may be due to its protection within fecal particles or adsorption to suspended solids [29,30].

As shown in Table 1, the absence of residual chlorine in the effluent of both hospitals correlated with the detection of SARS-CoV-2 RNA in wastewater. This persistence of viral RNA, even after chlorination, can be attributed to the high stability of RNA, which is often protected within fecal particles. Viruses, particularly those with sizes ranging from 1 to 200 nm, tend to adsorb onto suspended solids in wastewater, further stabilized by fat or protein layers. Within wastewater treatment plants (WWTPs), activated sludge acts as a microbial community composed of microorganisms and non-living organic and inorganic matter [31]. Enveloped viruses, such as coronaviruses, adsorb to solid materials in wastewater [32], which can shield them from disinfection by free chlorine [33,34]. For instance, Forés et al. (2021) reported that approximately 23% of coronaviruses in wastewater were attached to solid materials, highlighting their resistance to disinfection processes [35].

noted that the detection of viral RNA in wastewater is often associated with chemical oxygen demand (COD) and flow rates [34]. In this study, elevated levels of COD and biochemical oxygen demand (BOD) coincided with higher detection rates of SARS-CoV-2 RNA (Table 1). While biological processes like activated sludge can remove over 80% of BOD, COD, and total suspended solids (TSS), the presence of residual organic contaminants necessitates tertiary treatments such as chlorination, ozonation, and UV disinfection. Moreover, the persistence of antibiotic-resistant microorganisms, including viruses, often requires higher doses of disinfectants for effective inactivation [36].

In this study, SARS-CoV-2 RNA was detected in effluent samples when total and fecal coliform levels exceeded 1100 MPN/100 mL. The highest number of positive effluent samples was observed in May, coinciding with a surge in COVID-19 cases. This trend suggests that increased viral loads in influent wastewater can overwhelm treatment processes, leading to incomplete removal of viral RNA. In Razi Hospital, where the patient load was higher than in Imam Hospital, the elevated viral concentration in wastewater further challenged the treatment plant’s efficiency. Tandukar et al. (2022) similarly reported that high viral RNA concentrations in influent samples often correlate with peaks in COVID-19 transmission. [37]. Additionally, Serra-Compte et al. (2021) found SARS-CoV-2 RNA in both primary and secondary treatment stages, as well as in treated sludge after dewatering and anaerobic digestion, indicating that conventional treatment processes may not fully eliminate viral RNA. The tendency of enveloped viruses to adsorb to solid particles further contributes to their retention in sludge, as noted by Balboa et al. (2021). Consequently, the findings of this study suggest that WWTPs may release SARS-CoV-2 RNA into the environment, particularly in cases of high viral loads [38].

The high detection rate of SARS-CoV-2 RNA in hospital wastewater effluents underscores the potential of wastewater-based epidemiology (WBE) as a tool for disease surveillance and control. WBE can serve as an early warning system for COVID-19 outbreaks, especially in developing countries where wastewater treatment infrastructure may be inadequate [39]. However, it is important to note that the presence of viral RNA in wastewater does not necessarily indicate infectivity. Fecal marker-normalized SARS-CoV-2 RNA concentrations have been shown to correlate with clinical case numbers, suggesting that fecal indicators can help monitor fluctuations in viral loads and predict near-term trends in COVID-19 cases [37].

#### 3.1.2. Airborne transmission of SARS-CoV-2.

This study investigated the presence of SARS-CoV-2 RNA in hospital air, focusing on parameters such as temperature, relative humidity (RH), particulate matter (PM) concentrations, and distance from patients. Out of 23 air samples collected from Imam-Khomeini Hospital, 5 (21.74%) tested positive for SARS-CoV-2 RNA, with 4 positives in the COVID-19 patient ward and 1 in the ICU. In Razi Hospital, 7 out of 23 samples (30.43%) were positive, with detections in the COVID-19 ward, ICU, and CT scan areas. These findings align with Grimalt et al. (2022), who reported higher viral RNA concentrations in hospital corridors than in patient rooms, likely due to virus dispersion from rooms to common areas [2]. Stern et al. (2021) also found that non-COVID-19 wards occasionally showed high viral RNA levels, potentially reflecting asymptomatic infections among staff or broader community transmission [40]: The detailed results of SARS-CoV-2 RNA detection in hospital air samples are presented in Table 2.

**Table 2:** Details of Sampling Sites and Detection of SARS-CoV-2 in the air of Imam-Khomeini Hospital.

Hospital	No.	Sampling site		Exact location	Results	Temperature (ºC)	RH (%)	PM_10_^a^ (μg.m^-3^)	PM_2.5_^b^ (μg.m^-3^)	PM_1_^c^ (μg.m^-3^)
Imam-Khomeini	1	Special ward For COVID- 19 patient	Patient room	0.5 m	Positive	28	50	37.02	14.18	8.02
				1 m	Positive			32.16	10.05	7.35
				2 m	Positive			36.12	14.15	8.10
				5 m	Positive			32.09	10.08	7.35
			Nursing station	0.5 m	Negative	28	50	36.17	14.12	7.49
				1 m	Negative			34.27	13.27	8.01
				2 m	Negative			37.15	15.25	7.75
				5 m	Negative			36.02	14.75	7.01
	2	COVID-19 ICU	Patient room	0.5 m	Positive	24	45	29.02	10.11	7.30
				1 m	Negative			34.12	12.18	8.06
				2 m	Negative			28.12	10.28	7.40
				5 m	Negative			30.50	10.32	7.25
			Nursing station	0.5 m	Negative	22	55	27.01	8.75	7.01
				1 m	Negative			23.02	7.025	6.75
				2 m	Negative			26.12	8.62	7.01
				5 m	Negative			25.01	9.77	7.13
	3	CT scan ward	Location of Head	–	Negative	27	47	25.96	9.98	7.14
			Location of body	–	Negative			31.54	9.75	7.17
			1.5 m elevation	–	Negative			29.60	10.12	7.18
	4	Administrative Department ^d^	–	0.5 m	Negative	28	49	27.12	9.78	7.10
				1 m	Negative			26.76	9.93	7.14
				2 m	Negative			31.58	9.96	7.16
				5 m	Negative			25.61	10.15	7.16
Razi	1	Special ward For COVID- 19 patient	Patient room	0.5 m	Positive	27	52	30.25	10.48	8.14
				1 m	Positive			30.29	10.12	7.48
				2 m	Positive			32.08	10.30	7.39
				5 m	Negative			31.18	10.22	8.06
			Nursing station	0.5 m	Negative	25	47	32.16	10.11	7.38
				1 m	Negative			34.27	13.27	8.01
				2 m	Negative			37.15	15.25	7.75
				5 m	Negative			36.02	14.75	7.01
	2	COVID-19 ICU	Patient room	0.5 m	Positive	25	47	30.20	11.00	7.60
				1 m	Positive			30.48	10.28	7.12
				2 m	Negative			29.45	11.02	7.68
				5 m	Negative			42.05	11.39	8.68
			Nursing station	0.5 m	Negative	24	47	37.02	11.00	8.23
				1 m	Negative			23.02	7.025	6.75
				2 m	Negative			26.12	8.62	7.01
				5 m	Negative			25.01	9.77	7.13
	3	CT scan ward	Location of Head	–	Positive	27	47	39.02	10.80	8.02
			Location of body	–	Positive			36.08	9.85	6.78
			1.5 m elevation	–	Negative			42.12	11.20	8.78
	4	Administrative Department d	–	0.5 m	Negative	27	49	32.12	10.25	7.35
				1 m	Negative			38.07	11.15	7.89
				2 m	Negative			36.14	9.85	6.50
				5 m	Negative			40.15	10.48	8.56

**a) PM_10_ = PM < 10 μm; b) PM_2.5_ = PM < 2.5 μm; c) PM_1_ = PM < 1 μm; d) Samples were taken from the hospital’s Environmental Health unit.**

The detection of SARS-CoV-2 RNA in air samples underscores the importance of airborne transmission in healthcare settings. While close contact and respiratory droplets remain primary transmission routes, aerosols containing viral particles can travel beyond 2 meters, particularly in poorly ventilated spaces. This study detected viral RNA at distances of 0.5–5 meters from patients, consistent with findings by Lednicky et al. (2020) [17] and Santarpia et al. (2020) [41], who identified SARS-CoV-2 in airborne aerosols ≥2 meters from infected individuals. However, the results contrast with studies by Faridi et al. (2020) [42] and Masoumbeigi et al. (2020) [43], who reported no positive air samples, likely due to differences in ventilation systems or sampling methods. Effective ventilation and air filtration, such as HEPA systems, are critical to reducing airborne viral loads and mitigating transmission risks [44,45].

The study also measured PM concentrations (PM_10_, PM_2.5_, and PM_1_) in hospital air. PM_2.5_ and smaller particles (≤2.5 μm) are particularly concerning, as they can remain suspended in the air and carry viral particles, potentially depositing deep into the respiratory tract [46,47]. In Imam-Khomeini Hospital, PM_10_, PM_2.5_, and PM_1_ levels ranged from 23.02–37.15, 7.025–15.25, and 6.75–8.10 μg/m^3^, respectively. In Razi Hospital, PM levels were slightly higher, with PM_10_ reaching 42.12 μg/m^3^. While these values are below the WHO 24-hour average guidelines (PM_2.5_: 25 μg/m^3^; PM_10_: 50 μg/m^3^), they exceeded the stricter American Conference of Governmental Industrial Hygienists (ACGIH) standards for 8-hour exposure (PM_2.5_: 3 μg/m^3^; PM_10_: 10 μg/m^3^) [48]. Elevated PM levels in hospital wards may result from re-suspension of particles due to indoor activities, emphasizing the need for regular cleaning to minimize PM accumulation.

Studies have linked high PM_2.5_ concentrations to increased COVID-19 hospitalization and mortality rates [49–51]. Fine particles can carry viruses and exacerbate respiratory infections, reducing the body’s ability to combat pathogens [52]. Van Doremalen et al. (2020) demonstrated that SARS-CoV-2 remains viable on surfaces and in aerosols for extended periods, highlighting the role of PM in viral transmission [14].

Although this study found no direct correlation between temperature, RH, and viral RNA detection, other research suggests that low temperatures and low RH favor SARS-CoV-2 stability. For instance, Aboubakr et al. (2021) [53] and Matson et al. (2020) [54] reported that SARS-CoV-2 persists longer in cooler, drier conditions. At 20°C and 80% RH, the virus exhibited a half-life of 3 hours in aerosols [55]. Low RH can also compromise the respiratory tract’s defense mechanisms by drying mucus and impairing cilia function, increasing susceptibility to viral infections [56].

#### 3.1.3. Detection of SARS-CoV-2 on inanimate surfaces.

The study examined high-touch surfaces in Imam-Khomeini and Razi Hospitals, focusing on areas such as patient rooms, nursing stations, ICUs, CT scan wards, and administrative departments. Based on Table 3, a total of 92 surface samples were collected, with 26.09% (12/46) testing positive for SARS-CoV-2 RNA in Imam-Khomeini Hospital and 32.61% (15/46) in Razi Hospital. In Imam-Khomeini Hospital, positive samples were primarily found in the special ward (50%), ICU (25%), and CT scan ward (25%). In Razi Hospital, 66.66% of positive samples were from the special ward, 20% from the ICU, and 6.67% each from the CT scan and administrative areas. High-touch surfaces such as bed rails, bedside tables, computer keyboards, and medical equipment were frequently contaminated, highlighting the risk of fomite transmission.

**Table 3:** Details of Sampling Sites and Detection of SARS-CoV-2 on High-touch Surfaces of Imam-Khomeini Hospital.

Hospital	Sampling site	High-touch Environmental Surfaces		Result	Sampling site	High-touch Environmental Surfaces		Result
Imam-Khomeini	Special ward For COVID-19 patient	Patient room^*^ (T = 28°C; RH = 50%)	Doorknob	Negative	COVID-19 ICU	Patient (T = 24°C; RH = 45%)	Clothes	Positive
			Bed Rails	Positive			Bed Rails	Positive
			Bedside table	Positive			Bedside table	Negative
			Floor	Negative			Pillows	Positive
			Clothes	Positive			Floor	Negative
			Inside the refrigerator	Negative			Patient monitor	Negative
			Light switch	Negative		Nursing station (T = 26°C; RH = 46%)	Handles	Negative
			Garbage bin	Positive			Soaps tanks	Negative
			Cup	Negative			Alcohol tanks	Negative
			Curtain	Negative			Files	Negative
			Toilet sink	Negative			Cell phone	Negative
			Bed pillows	Positive	CT scan ward (T = 27°C; RH = 47%)	Doorknob		Negative
		Nursing station (T = 28°C; RH = 50%)	Handles	Negative		Location of the head		Positive
			Faucets	Negative		Location of the body		Negative
			Computer keyboards	Positive		Floor		Positive
			Soaps tanks	Negative		Wheelchair		Negative
			Alcohol tanks	Negative		Patient chair		Positive
			Blood pressure equipment	Negative	Administrative department^**^ (T = 28°C; RH = 49%)	Floor		Negative
			Files	Negative		Doorknob		Negative
			Pulse oximetry	Negative		Telephone		Negative
			Thermometers	Negative		Light switch		Negative
			Cell phone	Negative		Desk surface		Negative
			Air conditioner	Negative		Air conditioner		Negative
Razi	Special ward For COVID-19 patient	Patient room^*^ (T = 27°C; RH = 52%)	Doorknob	Negative	COVID-19 ICU	Patient (T = 24°C; RH = 45%)	Clothes	Positive
			Bed Rails	Positive			Bed Rails	Positive
			Bedside table	Positive			Bedside table	Negative
			Floor	Positive			Pillows	Positive
			Clothes	Positive			Floor	Negative
			Inside the refrigerator	Positive			Patient monitor	Negative
			Light switch	Negative		Nursing station (T = 26°C; RH = 46%)	Handles	Negative
			Garbage bin	Positive			Soaps tanks	Negative
			Cup	Positive			Alcohol tanks	Negative
			Curtain	Negative			Files	Negative
			Toilet sink	Negative			Cell phone	Negative
			Bed pillows	Positive	CT scan ward (T = 27°C; RH = 47%)	Doorknob		Negative
		Nursing station (T = 25°C; RH = 47%)	Handles	Negative		Location of the head		Negative
			Faucets	Negative		Location of the body		Negative
			Computer keyboards	Negative		Floor		Positive
			Soaps tanks	Negative		Wheelchair		Negative
			Alcohol tanks	Negative		Patient chair		Negative
			Blood pressure equipment	Positive	Administrative department^**^ (T = 28°C; RH = 49%)	Floor		Positive
			Files	Negative		Doorknob		Negative
			Pulse oximetry	Negative		Telephone		Negative
			Thermometers	Negative		Light switch		Negative
			Cell phone	Positive		Desk surface		Negative
			Air conditioner	Negative		Air conditioner		Negative

*Note: T = temperature; RH = relative humidity

** Samples were taken from the hospital’s Environmental Health unit.

Environmental conditions during sampling varied, with temperatures ranging from 24–28°C and relative humidity (RH) between 45–50% in Imam-Khomeini Hospital, and 24–27°C and 46–52% RH in Razi Hospital. These conditions, while not directly correlated with viral RNA detection in this study, are known to influence viral stability. For instance, higher temperatures and RH can reduce droplet size, facilitating longer airborne transmission [57,58].

The persistence of SARS-CoV-2 on high-touch surfaces underscores the importance of rigorous cleaning and disinfection protocols. Studies have shown that SARS-CoV-2 can survive on surfaces for hours to days, depending on material type and environmental conditions [59,60]. For example, Moore et al. (2021) [61]detected high viral RNA concentrations on door handles and bed rails, emphasizing the risk of hand-to-surface transmission. Jiang et al. (2020) [62] similarly found viral RNA on sheets, pillows, and counterpanes in quarantine rooms, reinforcing the need for frequent disinfection of high-touch surfaces.

In this study, contamination in ICUs and nursing stations was particularly concerning. The presence of viral RNA on patient clothing, pillows, and medical devices suggests that healthcare workers and visitors may inadvertently spread the virus if proper hygiene practices are not followed. Disposable gloves and personal protective equipment (PPE) are essential but must be used correctly to prevent surface contamination [63,64]. Regular glove changes and hand hygiene are critical to minimizing transmission risks.

The study revealed variability in surface contamination between the two hospitals. For instance, while SARS-CoV-2 RNA was detected on multiple surfaces in the CT scan ward of Imam-Khomeini Hospital, only the floor tested positive in Razi Hospital. This discrepancy may be attributed to differences in cleaning protocols or surface materials. Additionally, the absence of viral RNA in the administrative department of Imam-Khomeini Hospital suggests that strict hygiene measures, such as shoe covers and clothing changes, can effectively reduce contamination in non-clinical areas. However, the detection of viral RNA on the administrative floor in Razi Hospital highlights the need for consistent implementation of such measures.

The findings align with previous research demonstrating widespread surface contamination in healthcare settings. Bloise et al. (2020) [65] identified SARS-CoV-2 RNA on frequently touched objects like telephones and keyboards, underscoring the role of fomites in viral transmission. Similarly, Ding et al. (2021) [66]and Ye et al. (2020) [24] reported contamination in hospital wards, though they noted that widespread infection was unlikely if proper disinfection protocols were followed. These studies collectively emphasize the importance of targeted cleaning strategies, particularly in high-risk areas like ICUs and patient rooms.

### 3.2. Systematic review

To contextualize our case study findings within the broader scientific literature, we conducted a systematic review following PRISMA 2020 guidelines, synthesizing data from 47 studies on enveloped virus persistence in environmental matrices. This integrated approach allows us to compare our detection rates (wastewater: 65.28%; air: 21.74–30.43%; surfaces: 26.09–32.61%) with global data and identify consistent patterns as well as context-specific variations.

#### 3.2.1. Wastewater environment.

Wastewater-based epidemiology (WBE) has emerged as a valuable tool for monitoring viral outbreaks, particularly in healthcare settings. Based on Table 4, in our study, SARS-CoV-2 RNA was detected in 65.28% of wastewater samples collected from hospital WWTPs. Specifically, in Imam-Khomeini Hospital, 61.11% of influent samples tested positive for SARS-CoV-2 RNA, while only 11.11% of effluent samples were positive. Similarly, in Razi Hospital, 69.44% of influent samples were positive, compared to 16.67% of effluent samples. These findings align with Mohan et al. (2021), who demonstrated that enveloped viruses such as SARS-CoV-1 and HCoV-229E can remain viable in water for 2–100 days, depending on temperature, with longer survival at lower temperatures [67]. This confirms that temperature is a key factor in the persistence of viruses in aquatic environments. Additionally, La Rosa et al. (2020) reported that coronaviruses have low stability in water and are sensitive to oxidants like chlorine, which aligns with our observations of reduced viral RNA in effluent samples with residual chlorine [68]. Furthermore, Dalziel et al. (2016) showed that low pathogenic influenza A virus (LPAIV) can persist in water for hours to days, with factors such as temperature, pH, and salinity significantly influencing its survival. These results highlight the critical role of environmental conditions in the persistence of viruses in wastewater [69].

**Table 4:** Persistence and Detection of Viruses in Wastewater, Air and Surface: A Comprehensive Analysis.

Wastewater Environment					
Virus Type	Environment	Detection Method	Persistence Duration	Key Finding	References
SARS-CoV-1, HCoV-229E, TGEV, MHV, Phi6, MS2	Water	RT-qPCR, PCR, Culture-based	2 days to 100 days (depending on virus and temperature)	Viruses persist longer at lower temperatures; enveloped viruses inactivate faster than non-enveloped viruses.	[67]
Coronavirus (CoV)	Water (Wastewater, Surface Water, Drinking Water)	Real-time quantitative PCR, Metagenomics	Short (2–14 days depending on temperature)	CoV has low stability in water, sensitive to oxidants like chlorine, and is inactivated faster than non-enveloped viruses. No evidence of waterborne transmission in drinking water.	[68]
Coronavirus (CoV)	Water environments	RT-qPCR, PCR, Culture-based	2 days to 100 days (depending on virus and temperature)	CoV has low stability in the environment, is sensitive to oxidants like chlorine, and inactivates faster in water than non-enveloped viruses. Temperature significantly affects viral survival, with faster inactivation at higher temperatures. No evidence of human CoV in surface or groundwater. Further research is needed for concentration methods.	[68]
Low Pathogenic Influenza A Virus (LPAIV)	Water	Quantitative meta-analysis	Hours to days	Temperature, pH, salinity, and water type significantly affect virus persistence. Colder temperatures and unfiltered water increase persistence.	[69]
HIV-antiretrovirals (ARVs)	Surface water, WWTPs, Informal settlements	LC-QTOF/MS	Nevirapine: Stable; Lopinavir/Efavirenz: High persistence; Zidovudine: Degrades quickly	ARVs detected in water, posing risks to aquatic life. High concentrations linked to poor sanitation.	[83]
SARS-CoV-2	Wastewater, Hospital wastewater, Surface water	RT-PCR, RT-qPCR	Stable at pH 3–10; persists longer at 4°C	SARS-CoV-2 RNA detected in wastewater, indicating potential fecal-oral transmission. WBE useful for early outbreak detection. Advanced treatment methods like MBR are effective.	[70]
SARS-CoV-1, HCoV-229E, SARS-CoV-2, TGEV, MHV, Phi6, MS2	Wastewater	RT-qPCR, PCR, Culture-based	2 days to 33 days (depending on virus and temperature)	SARS-CoV-2 RNA persists in feces; enveloped viruses inactivate faster in wastewater.	[67]
SARS-CoV-2	Wastewater	RT-qPCR	2 days at 20°C, 14 days at 4°C	Detected in wastewater, indicating potential for monitoring community spread through wastewater surveillance.	[72]
SARS-CoV-2	Wastewater, surface water, sludge	RT-PCR, metagenomic analysis, biosensor technology	Several days in untreated wastewater; up to 7 days in tap water; 14 days at 4°C	SARS-CoV-2 RNA detected in wastewater globally; no evidence of transmission via water, but potential for faecal-oral transmission exists. Coagulation, filtration, and chlorination can remove/inactivate the virus.	[73]
SARS-CoV	Wastewater, Water	RT-PCR, RT-qPCR	Up to 14 days at 4°C	Detected in wastewater; persistence influenced by pH, temperature, and composition.	[70]
SARS-CoV-2	Wastewater	RT-qPCR, Genomic Sequencing	Hours to days	Detected year-round; higher concentrations in winter. Sequencing detected lower abundances, while RT-qPCR provided accurate quantification.	[71]
Influenza A/B	Wastewater	RT-qPCR, Genomic Sequencing	Hours to days	Detected in 75% of samples via sequencing; higher detection in winter.	[71]
Enterovirus	Wastewater	RT-qPCR, Genomic Sequencing	Hours to days	Detected in all samples via sequencing; only one sample positive via RT-qPCR.	[71]
Air and Surface Environment					
Virus Type	Environment	Detection Method	Persistence Duration	Key Finding	References
SARS-CoV-2	Hospital settings, ICU vs Non-ICU, Toilets/Bathrooms, Clinical Areas, Staff Areas, Public Areas, Close Patient Environments, PPE Removal Rooms, Medical Staff Offices	RT-PCR, Viral Culture	Not specified	*17.4% of air samples near patients were positive for SARS-CoV-2 RNA. Higher positivity rate in ICU (25.2%) compared to non-ICU (10.7%). *23.8% of air samples in toilets/bathrooms were positive for SARS-CoV-2 RNA. *8.4% of air samples in clinical areas were positive for SARS-CoV-2 RNA. - 12.3% of air samples in staff areas were positive for SARS-CoV-2 RNA. *33.3% of air samples in public areas were positive for SARS-CoV-2 RNA. *7 out of 47 viral cultures were positive, all from close patient environments. *High concentrations of SARS-CoV-2 RNA found in PPE removal rooms. *Peaks in particle size greater than 4 μm in medical staff offices.	[74]
Influenza A, Influenza B, Rhinovirus, RSV, Human Coronavirus	Air (University Campus)	rRT-qPCR	Not specified	*Influenza A detected at 16.9% frequency. *Influenza B detected at 4.5% frequency. *Rhinovirus detected at 2.2% frequency. *RSV detected at 0.4% frequency. * Human coronaviruses (229E/OC43) not detected.	[76]
Influenza A, B, RSV	Air (Urgent Care Clinic)	Real-time quantitative PCR	Extended time (particles ≤4.1 μm remain airborne)	Airborne RNA detected; Influenza A and RSV widespread, especially during peak patient loads; RSV more widely distributed than influenza.	[77]
RSV (Respiratory Syncytial Virus)	Pediatric Acute Care Clinic	2-stage bioaerosol cyclone samplers + real-time PCR	Not specified	Airborne RSV detected in 2.3% of samples; most particles ≥4.1 µm, suggesting inefficient airborne transmission. Masks and goggles may not be necessary in outpatient settings.	[78]
SARS-CoV-2	Air, surfaces, wastewater	Real-time RT-PCR	Hours to days	29% of air, 16% of surface, and 37.5% of wastewater samples were positive.	[80]
Influenza Virus	Air, Water, Feces, Fomites	Systematic review of persistence studies	Variable (depends on matrix and conditions)	Influenza virus persists longer in water than in air; temperature, salinity, and pH significantly affect persistence. Low temperatures and normal pH increase persistence, while high salinity reduces it.	[81]
Cytomegalovirus (CMV)	Hospital rooms of immunocompromised patients	PCR (Polymerase Chain Reaction)	Not specified (detected during 6-hour air sampling)	CMV-DNA was detected in the air of patients with active CMV pneumonia and a latent CMV infection, suggesting potential for airborne transmission in immunocompromised populations.	[82]
SARS-CoV-2	Hospital rooms (ICU and wards)	Air and surface sampling using NIOSH bioaerosol samplers and PCR for RNA detection	Up to 3 hours in air	SARS-CoV-2 RNA detected in air and on surfaces, especially in the first week of illness. High-touch surfaces and floors were most contaminated. Airborne particles were detected in sizes >4 μm and 1–4 μm.	[84]
SARS-CoV-2	Hospital isolation wards and ICU	Air and surface sampling using RT-PCR for RNA detection	Up to 72 hours on surfaces, 3 hours in air	Environmental contamination with SARS-CoV-2 RNA was relatively low, but higher on high-touch surfaces near severe/critical patients. Airborne contamination was detected during intubation procedures.	[75]
SARS-CoV-2	Hospital rooms (negative pressure bays and isolation rooms)	Real-time RT-PCR for RNA detection	Up to 3 days in aerosols (experimental)	Environmental contamination by SARS-CoV-2 was low, detected on stethoscopes, intubation tubes, and PPE. No airborne contamination was detected, possibly due to negative pressure environments.	[85]
SARS-CoV-2	Hospital COVID-19 ward (contaminated, semi-contaminated, and clean areas)	Real-time RT-PCR for RNA detection	Up to 72 hours on surfaces, 3 hours in air (experimental)	SARS-CoV-2 RNA was detected in air and on surfaces in contaminated and semi-contaminated areas, but not in clean areas. High-touch surfaces and medical equipment were most contaminated. Airborne transmission was observed in the ICU and patient corridors.	[86]
Adenovirus, Herpes Simplex Virus, Cytomegalovirus, Vaccinia Virus, Rotavirus, Coxsackie Virus, Poliovirus, Rhinovirus, Hepatitis A Virus, Caliciviridae, Hepatitis C Virus, Influenza A/B, Parainfluenza Virus, RSV, Ebola Virus, SARS-CoV-1, SARS-CoV-2, HIV, Hepatitis B Virus	Surfaces (glass, plastic, aluminum, stainless steel, paper, cloth, plexiglass, cotton, latex gloves, Formica, ceramics, cardboard, copper, syringe needles, banknotes, petri dishes, silanized tubes)	Infectivity assay, Antigen detection	4.5 hours– > 12 weeks (Adenovirus: > 12 weeks; HSV: 4.5 hours– > 8 weeks; CMV: up to 8 hours; Vaccinia: up to 8 weeks; Rotavirus: 2– > 60 days; Coxsackie: up to 5 weeks; Polio: 1 day–60 days; Rhinovirus: up to 24 hours; HAV: 2 hours– > 60 days; Norovirus: 8 hours– > 168 days; HCV: 5 days– > 6 weeks; Influenza A: 6 hours–2 weeks; Influenza B: 8–48 hours; Parainfluenza: 2– > 8 hours; RSV: 0.5–7 hours; Ebola: 2–48 days; SARS-CoV-1: 24 hours–8 days; SARS-CoV-2: 4 hours– > 8 days; HIV: up to 7 days; HBV: > 14 days)	Low humidity and temperature increase survival for most viruses. Copper surfaces inactivate some viruses quickly. SARS-CoV-2 has biphasic decay. Fecal suspension can protect some viruses. High RH reduces survival for many viruses.	[79]

In our study, SARS-CoV-2 RNA was detected in 65.28% of hospital wastewater samples, consistent with the findings of Adelodun et al. (2021). They reported that SARS-CoV-2 RNA in wastewater indicates the potential for fecal-oral transmission [70]. Similarly, Mohan et al. (2021) demonstrated that SARS-CoV-2 RNA persists in feces, and enveloped viruses inactivate faster in wastewater than non-enveloped viruses. These findings emphasize that hospital wastewater can serve as an important source for monitoring disease outbreaks [67]. Moreover, Williams et al. (2024) reported that SARS-CoV-2 RNA is detected in wastewater year-round, with higher concentrations in winter [71], which aligns with our observations of increased viral loads during colder periods.

The persistence of SARS-CoV-2 in wastewater has been further elucidated by studies such as Corpuz et al. (2020), who found that the virus can remain detectable for 2 days at 20°C and up to 14 days at 4°C, underscoring the potential of wastewater surveillance for monitoring community spread [72]. Similarly, Mohapatra et al. (2021) highlighted that SARS-CoV-2 RNA can persist for several days in untreated wastewater and up to 7 days in tap water, with coagulation, filtration, and chlorination proving effective in removing or inactivating the virus [73].

In addition to SARS-CoV-2, other viruses such as influenza and enteroviruses have also been detected in wastewater. Williams et al. (2024) found that influenza RNA was detected in 75% of wastewater samples, with higher detection rates in winter. Enteroviruses were detected in all wastewater samples, although only one sample tested positive using RT-qPCR [71]. These findings indicate that wastewater can serve as a valuable tool for monitoring the spread of various viruses. In conclusion, integrating wastewater surveillance with advanced treatment methods, such as membrane bioreactors (MBR), is essential for mitigating the risks associated with viral persistence in aquatic environments and preventing environmental transmission.

#### 3.2.2. Air and surface environment.

The persistence and detection of enveloped viruses in air and surface environments, particularly in healthcare settings, have been extensively studied. In this section, the findings from the case study conducted at Imam Khomeini and Razi Hospitals are compared with the results of a systematic review to provide a comprehensive understanding of the persistence of these viruses in various environments.

##### 3.2.2.1. Airborne transmission of enveloped viruses:

In our study, RNA of enveloped viruses, such as SARS-CoV-2, was detected in 21.74% of air samples from Imam Khomeini Hospital and 30.43% from Razi Hospital. These results align with the findings of Birgand et al. (2020), who reported that enveloped viruses like SARS-CoV-2 can remain viable in the air, with a higher risk of transmission in poorly ventilated spaces [74]. The study by Chia et al. (2020a) also confirms that airborne particles containing enveloped viruses can travel more than 2 meters, which is consistent with the case study’s observation of viral RNA detection at distances of 0.5 to 5 meters from patients. This highlights the importance of effective ventilation systems, such as HEPA filters [8]. The study by Tan et al. (2020) further supports this, showing that airborne contamination is more prevalent during aerosol-generating procedures, such as intubation, which aligns with the case study’s findings of higher viral RNA concentrations in ICU settings [75].

In addition to SARS-CoV-2, other enveloped viruses, such as influenza viruses and respiratory syncytial virus (RSV), can also be transmitted through the air. The study by Xie et al. (2020) demonstrated that influenza A virus was detected in 16.9% of air samples from university environments, while influenza B and RSV were detected in 4.5% and 0.4% of samples, respectively. These findings indicate that various enveloped viruses can remain viable in the air, increasing the risk of transmission [76]. Similarly, Lindsley et al. (2010) found that airborne RNA of influenza A and RSV was widespread in urgent care clinics, especially during peak patient loads, with RSV being more widely distributed than influenza [77]. Grayson et al. (2017) also detected airborne RSV in pediatric acute care clinics, with most particles ≥4.1 µm, suggesting inefficient airborne transmission and raising questions about the necessity of masks and goggles in outpatient settings [78].

##### 3.2.2.2. Surface contamination by enveloped viruses:

In our study, 26.09% of surface samples from Imam Khomeini Hospital and 32.61% from Razi Hospital tested positive for enveloped viruses. High-touch surfaces such as bed rails, bedside tables, and medical equipment were the most frequently contaminated. These results are consistent with the findings of Moore et al. (2021), who reported high concentrations of viral RNA on door handles and bed rails [61]. Similarly, Jiang et al. (2020) found viral RNA on sheets, pillows, and countertops in quarantine rooms, emphasizing the need for thorough cleaning and disinfection of high-touch surfaces. The persistence of enveloped viruses on surfaces is influenced by temperature and humidity [62]. In the case study, surface contamination was observed in environments with temperatures ranging from 24–28°C and relative humidity (RH) of 45–50%. These conditions align with the findings of Wißmann et al. (2021), who reported that low humidity and low temperatures increase the survival of viruses [79]. The study by Liu et al. (2020) also showed that higher temperatures and relative humidity may reduce droplet size and facilitate longer airborne transmission [57].

The persistence of enveloped viruses varies depending on the environment and conditions. For instance, Ziarani et al. (2022) found that 29% of air, 16% of surface, and 37.5% of wastewater samples tested positive for SARS-CoV-2 RNA, highlighting the potential for environmental transmission [80]. Similarly, studies on influenza viruses have shown that they persist longer in water than in air, with temperature, salinity, and pH significantly affecting their survival [81]. Low temperatures and normal pH increase persistence, while high salinity reduces it. Cytomegalovirus (CMV), another enveloped virus, has also been detected in the air of hospital rooms housing immunocompromised patients, suggesting potential for airborne transmission in vulnerable populations [82].

## 4. Limitations of the study

Several limitations should be considered when interpreting our findings. First, RT-PCR detects viral RNA but does not distinguish between infectious and non-infectious particles; therefore, the presence of SARS-CoV-2 RNA does not necessarily indicate viable virus or transmission risk. Second, this study was designed as exploratory environmental surveillance with descriptive objectives, and inferential statistical analyses (e.g., correlation tests) were not performed due to the qualitative nature of the data and limited subgroup sample sizes. Third, wastewater samples were collected using grab sampling rather than 24-hour composite sampling, which may not fully capture daily fluctuations in viral shedding. Fourth, the study was conducted in two hospitals in northern Iran during a specific period (May-August 2022), which may limit generalizability to other geographic regions or seasons. Despite these limitations, this study provides valuable real-world data on the persistence of enveloped viruses in hospital environments and offers important implications for infection control practices.

## 5. Conclusion

This study provides a thorough examination of enveloped viruses, with a specific focus on the persistence of SARS-CoV-2 in hospital environments. Through an integrated analysis of wastewater, air, and surface samples, complemented by a systematic literature review, the research offers critical insights into viral dynamics within healthcare settings. The findings indicate a significant presence of viral RNA, with detection in 65.28% of wastewater samples (47 out of 72), suggesting considerable viral shedding from infected patients. Notably, higher viral loads were found to correlate with increased levels of chemical oxygen demand and coliform counts in the wastewater. Additionally, the analysis of air samples revealed SARS-CoV-2 RNA in 26.09% of cases (12 out of 46), primarily sourced from COVID-19 wards and intensive care units (ICUs), with detectable RNA present up to 5 meters from the patients. The association between SARS-CoV-2 RNA and elevated PM2.5 and PM10 levels, despite remaining below the thresholds outlined by WHO, highlights a pressing need for enhanced ventilation strategies in clinical environments to reduce the risk of airborne transmission. Surface contamination analysis demonstrated variability in positivity rates across different hospitals (26.09% vs. 32.61%), particularly in high-touch areas such as bed rails and medical equipment. This underscores the importance of maintaining strict adherence to established cleaning protocols to effectively manage viral persistence on surfaces. Overall, the findings underscore the necessity for comprehensive studies on enveloped viruses and their environmental persistence. Key recommendations arising from this research include: implementing routine wastewater monitoring as a proactive early warning system, optimizing air quality management in high-risk areas, and standardizing stringent surface disinfection protocols. While the detection of RNA in environmental samples confirms viral presence, it is crucial to note that this does not imply infectivity, necessitating further studies to correlate these environmental findings with clinical transmission data.

## Supporting information

S1 Table Summary of quality assessment for all included studies. (DOCX)
