# Supplementary material for: Integrated monitoring of enveloped viruses in hospital environments: Detection, persistence, and implications for infection control
Source: PLoS One. 2026 Apr 30;21(4):e0345644. doi: 10.1371/journal.pone.0345644 (PMC13132218; doi:10.1371/journal.pone.0345644)

**Table S1: Quality Assessment of Articles in Systematic Review on Integrated Monitoring of Enveloped Viruses**

| First author | Year | Title | Journal | CiteScore Quartile | Ref. |  |  |
| --- | --- | --- | --- | --- | --- | --- | --- |
| Mohan SV | 2021 | SARS-CoV-2 in environmental perspective: Occurrence, persistence, surveillance, inactivation and challenges | Chemical Engineering Journal | Q1 | **67** |  |  |
| La Rosa G | 2020 | Coronavirus in water environments: Occurrence, persistence and concentration methods - A scoping review | Water Research | Q1 | **68** |  |  |
| Dalziel AE | 2016 | Persistence of low pathogenic influenza A virus in water: a systematic review and quantitative meta-analysis | PLoS One | Q2 | **69** |  |  |
| Adelodun B | 2021 | Presence, detection, and persistence of SARS-CoV-2 in wastewater and the sustainable remedial measures | Environmental and Health Management of Novel Coronavirus Disease (COVID-19) (Book Chapter) | — | **70** |  |  |
| Williams RC | 2024 | Simultaneous detection and characterization of common respiratory pathogens in wastewater through genomic sequencing | Water Research | Q1 | **71** |  |  |
| Corpuz MVA | 2020 | Viruses in wastewater: occurrence, abundance and detection methods | Science of the Total Environment | Q1 | **72** |  |  |
| Mohapatra S | 2021 | The novel SARS-CoV-2 pandemic: possible environmental transmission, detection, persistence and fate during wastewater and water treatment | Science of the Total Environment | Q1 | **73** |  |  |
| Birgand G | 2020 | Assessment of air contamination by SARS-CoV-2 in hospital settings | JAMA Network Open | Q1 | **74** |  |  |
| Tan L | 2020 | Air and surface contamination by SARS-CoV-2 virus in a tertiary hospital in Wuhan, China | International Journal of Infectious Diseases | Q1 | **75** |  |  |
| Xie C | 2020 | Detection of influenza and other respiratory viruses in air sampled from a university campus: a longitudinal study | Clinical Infectious Diseases | Q1 | **76** |  |  |
| Lindsley WG | 2010 | Distribution of airborne influenza virus and respiratory syncytial virus in an urgent care medical clinic | Clinical Infectious Diseases | Q1 | **77** |  |  |
| Grayson SA | 2017 | Detection of airborne respiratory syncytial virus in a pediatric acute care clinic | Pediatric Pulmonology | Q1 | **78** |  |  |
| Wißmann JE | 2021 | Persistence of pathogens on inanimate surfaces: a narrative review | Microorganisms | Q2 | **79** |  |  |
| Ziarani FR | 2022 | Detection of SARS-CoV-2 genome in the air, surfaces, and wastewater of the referral hospitals, Gorgan, north of Iran | Iranian Journal of Microbiology | Q3 | **80** |  |  |
| Irwin CK | 2011 | Using the systematic review methodology to evaluate factors that influence the persistence of influenza virus in environmental matrices | Applied and Environmental Microbiology | Q1 | **81** |  |  |
| McCluskey R | 1996 | Detection of airborne cytomegalovirus in hospital rooms of immunocompromised patients | Journal of Virological Methods | Q2 | **82** |  |  |
| Horn S | 2022 | HIV-antiretrovirals in river water from Gauteng, South Africa: Mixed messages of wastewater inflows as source | Science of the Total Environment | Q1 | **83** |  |  |
| Chia PY | 2020 | Detection of air and surface contamination by SARS-CoV-2 in hospital rooms of infected patients | Nature Communications | Q1 | **84** |  |  |
| Nakamura K | 2020 | Environmental surface and air contamination in severe acute respiratory syndrome coronavirus 2 (SARS-CoV-2) patient rooms by disease severity | Infection Prevention in Practice | Q2 | **85** |  |  |
| Razzini K | 2020 | SARS-CoV-2 RNA detection in the air and on surfaces in the COVID-19 ward of a hospital in Milan, Italy | Science of the Total Environment | Q1 | **86** |  |  |


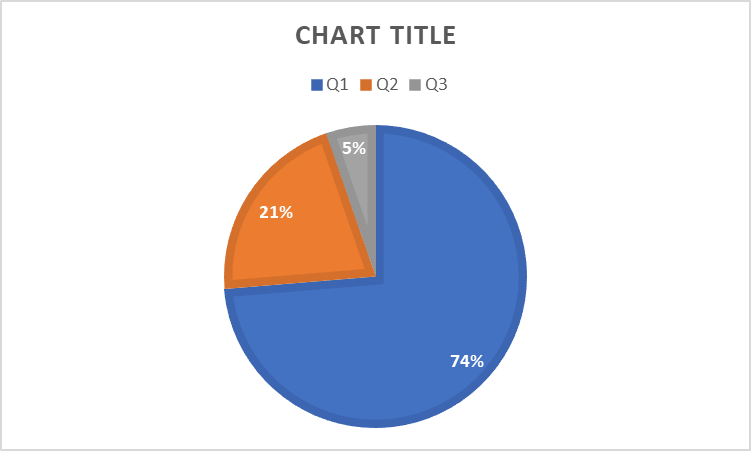

Supplement: S1 Table — (DOCX) [file pone.0345644.s001.docx]
